# Creativity in bipolar disorder: a systematic review

**DOI:** 10.47626/2237-6089-2021-0196

**Published:** 2022-10-18

**Authors:** Thiara Nascimento da Cruz, Evelyn V. Camelo, Antonio Egidio Nardi, Elie Cheniaux

**Affiliations:** 1 Instituto de Psiquiatria Universidade Federal do Rio de Janeiro Rio de Janeiro RJ Brazil Instituto de Psiquiatria (IPUB), Universidade Federal do Rio de Janeiro (UFRJ), Rio de Janeiro , RJ , Brazil .; 2 UFRJ Rio de Janeiro RJ Brazil UFRJ , Rio de Janeiro , RJ , Brazil .; 3 Universidade do Estado do Rio de Janeiro Rio de Janeiro RJ Brazil Universidade do Estado do Rio de Janeiro (UERJ), Rio de Janeiro , RJ , Brazil .

**Keywords:** Bipolar disorder, creativity, systematic review

## Abstract

**Introduction:**

Based on studies of the biographies of artists and on research in which modern diagnostic criteria were applied, it has been suggested that there is a relationship between bipolar disorder (BD) and creativity. Objective: To investigate the relationship between BD and creativity and whether creative capacity varies depending on mood state.

**Method:**

We conducted a systematic search of the scientific literature indexed on the PubMed, ISI Web of Science, PsycINFO, and SciELO databases using the terms “bipolar” OR “bipolar disorder” OR “mania” OR “manic” AND “creativ*”. Original studies were selected that investigated samples of at least ten patients with BD using at least one psychometric instrument to assess creativity.

**Results:**

Twelve articles met the selection criteria. The results of comparisons of BD patients with control groups without BD were heterogeneous. BD was not associated with higher levels of creativity than other mental disorders. When comparing BD phases, depression was associated with worse performance on creativity tests and patients in mania (or hypomania) were not distinguished from euthymia patients.

**Conclusion:**

It was not possible to corroborate the hypothesis that individuals with BD are more creative than individuals without psychiatric diagnoses or than patients suffering from other mental disorders, which may be related to the cross-sectional rather than longitudinal designs of virtually all of the clinical studies.

## Introduction

Bipolar disorder (BD) is a serious, chronic, and disabling mental disorder characterized by the occurrence of hypomanic or manic episodes and depressive episodes. ^1^ After the first episode of the disease, almost 90% of individuals experience at least one further episode throughout their lives. ^2^ Many individuals have progressive cognitive impairment. ^3^ Between 30 and 60% of BD patients have low psychosocial functioning. ^4^ Compared to the general population, BD patients have higher unemployment rates and lower income and, when employed, spend more time on sick leave. ^5^ BD is the mental disorder most associated with suicide. Approximately 40% of patients attempt suicide at least once in their lives. ^6^ The rate of completed suicides can be up to 58 times higher in BD patients than in the general population and these suicides are violent in more than 90% of cases. ^7^

Despite the severity of BD, many authors suggest that there are positive aspects to the disease. Authors such as psychologist Kay R. Jamison and psychiatrists Nancy Andreasen and Arnold K. Ludwig believe that there is a relationship between BD and creativity, based on studies they have conducted of the biographies of eminently creative artists and writers. ^8 - 11^ Moreover, studies using modern diagnostic criteria have found higher prevalence of depressive and manic episodes in artists and writers, compared to the general population. ^12^

There are many definitions of creativity, but none are universally accepted. ^13^ Based on the “Handbook of Creativity,” ^14^ the most mentioned publication on the topic, creativity is the ability to combine talent, process, and environment to produce something that is perceived as original and, at the same time, useful in the context. ^15^ Creativity is a multidimensional skill demonstrated by a person, through a process, which creates a product, in response to the environment in which it operates. ^16 - 18^

Studies of the creative person seek to evaluate individual characteristics that favor creativity, such as intrinsic motivation, openness to experience, variety of interests, and autonomy. ^17^ Creative people stand out for their perception of problems, fluency of ideas, mental flexibility, divergent thinking, ability to redefine familiar objects and concepts, positive mood, complex temperament, and self-confidence. ^19^ Creativity can be assessed using personality scales, derived from the study of common characteristics of people already considered creative, or by researching previous behaviors that may reflect creative potential and accomplishments. ^17^ The following instruments, respectively, are examples of these two possibilities: the Adjective Check List Creative Personality Scale (ACL-CPS) ^20^ and the Creativity Achievement Questionnaires (CAQ). ^21^ People can also be assessed for creativity in a specific field of activity, such as in the fields of linguistics, logic, arts, or sciences. ^22^ The specific area most investigated is that of artistic creativity, most commonly using the Barron-Welsh Art Scale (BWAS). ^23 , 24^ This scale comprises figures of different degrees of complexity and varying levels of symmetry. Subjects indicate whether they like or dislike each image. Preference for high complexity and asymmetry is considered an indicator of creativity. ^25^

Studies of the creative process seek to understand the mental mechanism involved when an individual performs a creative activity. ^17^ This aspect of creativity can be evaluated based on the results of tests of divergent thinking and remote association. These tests use criteria based on originality, usefulness, and rarity of responses. Divergent thinking tests are more widely used and the Torrance Tests of Creative Thinking (TTCT) is considered the gold standard. ^26^

Creative product reviews focus on something a person does or expresses, such as poems, music, drawings, or answers to an open question. Just as a person can be evaluated in a specific field of activity, their creative product can also be classified in the same way. ^27^ This aspect is usually analyzed according to the opinion of experts, evaluation of teachers and peers, eminence, and self-reported creative activities and achievements. ^18^ The Lifetime Creativity Scales (LCS) ^28^ is an example of an instrument that evaluates the creative product, measuring the quantity and quality of a person’s involvement in creative activities throughout adult life, both in free time, and at work. Finally, assessments of the creative environment seek to find characteristics that induce or inhibit creativity. The environment is rarely considered during evaluation of individual creativity. ^27^ The Virtual Team Creative Climate Measure (VTCC) ^29^ is an example of such an instrument, evaluating items such as acceptance of ideas, collaboration, commitment, and clarity of objectives, among others. ^17^

Research on creativity can result in many controversies, mainly because of weaknesses in the methodologies used. ^27^ The first problem resides in the concept of creativity. Few definitions are widely accepted and researchers often avoid using a definition. ^17^ The second problem is the proper choice of instruments for the research objectives. It is essential to distinguish between creative potential and creative achievements. An instrument capable of predicting creative potential is very different from another that measures current levels of performance. ^26^ The format of studies constitutes a third problem. Although more difficult to accomplish, the most reliable form of research is longitudinal. ^17^ Longitudinal studies can evaluate the creative capacity of individuals who, for a variety of reasons, may not yet have achieved exceptional results. ^30^ Finally, the fourth problem is the tendency to investigate only one aspect of the concept of creativity, which is multidimensional. Psychometric instruments generally only assess one of the four aspects of creativity – person, process, product, or environment – in isolation. ^17^ Since each instrument has its limitations, it is highly recommended to select a combination of instruments. ^26^

At least two systematic reviews ^31 , 32^ have studied the relationship between psychopathology and creativity. Thys et al. ^31^ found an association between suffering from a mental disorder, especially a mood disorder or a psychotic disorder, and being creative. However, the studies included in this review were highly heterogeneous and most of them had important methodological limitations. In addition, their conclusions are based on a mere count of the number of articles that presented positive or negative results on whether a certain mental disorder was related to creativity. In contrast, Taylor ^32^ carried out a meta-analysis and was more rigorous in its selection of the original studies. According to her results, there were more cases of mood disorders among individuals considered to be creative than among non-creative individuals. On the other hand, when individuals with mood disorders were compared with others without this diagnosis, no significant differences were found regarding the level of creativity. Neither of these two systematic reviews specifically addressed BD.

We conducted a systematic review of clinical studies that objectively assessed creativity in patients with BD in order to investigate the relationship between this mental disorder and creativity and whether creative capacity varies depending on BD mood state, i.e., mania, depression, or euthymia.

## Method

We sought studies in which patients with BD were compared in terms of creative ability with healthy controls or with patients with other mental disorders. We also sought studies with samples of patients with BD in which the different states of the disease – mania, depression, mixed state, and euthymia – were compared.

Up to January of 2021, we carried out a systematic search of the scientific literature indexed on the PubMed, ISI Web of Science, PsycINFO, and SciELO databases using the terms “bipolar” OR “bipolar disorder” OR “mania” OR “manic” AND “creativ*”. There was no restriction on the date of publication. Articles that met the following criteria were selected: original studies, published in Portuguese or English, with samples of at least 10 BD patients, and using at least one psychometric instrument to assess creativity. The diagnosis of BD should be made using operational criteria. Letters, editorials, review articles, case studies, and biographical studies were excluded. Two of the authors carried out each step of this search independently, compared their results and reached a consensus. We used the Preferred Reporting Items for Systematic Reviews and Meta-Analyses (PRISMA) flowchart to illustrate the search strategy adopted to select the articles. ^33^

## Results

**Figure 1:**
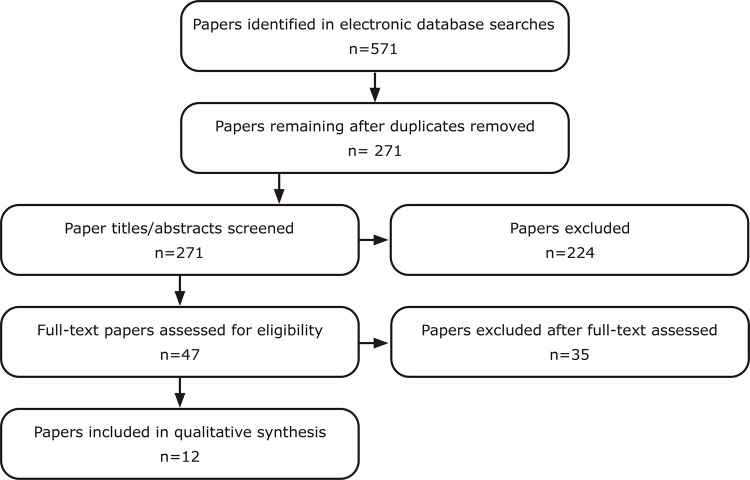
Systematic search according to the Preferred Reporting Items for Systematic Reviews and Meta-Analyses (PRISMA) Statement

We found 165 results in the PubMed database, 179 in ISI Web of Science, 222 in PsycINFO, and only five in SciELO, making a total of 571 articles. After removing the duplicates, 271 articles remained, 224 of which were excluded during reading of titles and abstracts. After reading the remaining 47 articles in full, some were excluded: nine because the subjects were not diagnosed with BD; nine in which there was no objective evaluation of creativity; six because they were letters or editorials; six that were case studies or biographical studies; three in which the authors evaluated aspects related to creativity, but not creativity itself; and one review article. We were unable to access one article, even after trying to contact the authors directly. Thus, 12 articles were selected according to our criteria. ^34 - 45^ Our systematic search method is illustrated as a flowchart in Figure 1 .

Considering the 12 studies selected, the samples were quite heterogeneous. Five studies evaluated only type I BD ^34 , 36 , 38 - 40^ and the others used mixed samples of type I, type II, and unspecified BD. Two studies analyzed patients who were not using medication, ^36 , 40^ two studies included both medicated and non-medicated subjects, ^39 , 42^ and the others used samples in which all patients were using medication. The groups involved men and women with ages ranging from 8 to 67 years old. Sample sizes for BD patients ranged between 17 and 397. Most studies tested at least 60 patients. None of the studies carried out a comparison between BD I and BD II.

In the 12 studies selected, BD was diagnosed according to the Diagnostic and Statistical Manual of Mental Disorders, 4th edition (DSM-IV) criteria, ^46^ or by application of the Structured Clinical Interview for DSM Diagnosis (SCID), ^47^ except for one study ^45^ that used the DSM-II and DSM-III criteria, ^48 , 49^ based on data from patients’ medical records. Ten studies used clinical mania rating scales: the Young Mania Rating Scale (YMRS) ^50^ in eight studies and the Altman Self-Rating Mania Scale (ASRM) ^51^ in two. ^37 , 38^ The Montgomery-Asberg Depression Rating Scale (MADRS) ^52^ was used to evaluate depression in three studies ^36 , 39 , 40^ ; the Hamilton Depression Rating Scale (HDRS-17) ^53^ was used in three ^34 , 35 , 41^ ; the Beck Depression Inventory (BDI) ^54^ was used in two ^38 , 42^ ; the Modified Hamilton Rating Scale for Depression (MHRSD) ^55^ ; the Depression Anxiety Stress Scale (DASS-21) ^56^ ; the Children’s Depression Rating Scale-Revised (CDRS-R) ^57^ ; the 10-Item Center for Epidemiological Studies Depression Scale (CESD-10) ^58^ ; and the Rosenberg Self-esteem Scale (RSS) ^59^ were each used in one study. ^37 , 38 , 43 , 44^

The 12 studies selected researched different aspects of creativity, so they used different methods. In total, 15 instruments were used. The number varied from one to four instruments per study. Five studies only used instruments to assess characteristics of the creative person, ^36 , 37 , 39 , 40 , 43^ three studies only evaluated the creative process, ^34 , 35 , 44^ three used instruments to assess both personal characteristics and the creative process, ^38 , 41 , 42^ and only one study evaluated the creative product. ^45^ All studies, except one, ^45^ had cross-sectional designs.

The scale most used to investigate the characteristics of the creative person was the BWAS, ^23 , 24^ which was applied in five ^36 , 39 , 40 , 42 , 43^ of the 12 studies and was the only instrument in four studies. ^36 , 39 , 40 , 43^ One study ^41^ used a reduced version of the BWAS, the Revised Art Scale (RAS). ^60^ The other instruments used to evaluate people’s characteristics, each used in only one study, were the ACL-CPS, ^20 , 42^ the Creative Achievement Questionnaire (CAQ), ^21 , 38^ and the Creativity Domain Questionnaire-Revised (CDQ-R). ^37 , 61^ The creative process was evaluated using the Alternate Uses Test (AuT), ^62^ the Abbreviated Torrance Test for Adults (ATTA), ^63^ the Remote Associates Test (RAT), ^64^ and the Chinese Word Remote Associates Test (CWRAT - the Chinese version of the RAT), ^65^ each used in two studies, ^35 , 38^ while the Unusual Uses Test (UUT), ^38 , 66^ the inventiveness battery of the Berlin Intelligence Structure Test (BIS), ^41 , 67^ the TTCT-Figural (TTCT-F) and TTCT-Verbal (TTCT-V) versions, ^42 , 68^ and the Creative Thinking Task – designed specifically for the study, ^44^ were each used in only one study. Only one study evaluated the creative product, ^45^ using the LCS. ^28^ The creative environment was not evaluated in any studies.

Ten of the 12 studies compared BD patients with healthy individuals, with individuals with other mental disorders, or with both ^34 , 35 , 38 - 45^ ( Tables 1 and 2 ). Four of these 10 studies only selected euthymic patients, ^34 , 35 , 38 , 42^ while the others included subjects in different mood states.

**Table 1:** Studies comparing BD patients with healthy controls

Study	Design and samples	Clinical assessment instruments	Creativity assessment instruments	Results
Alici ^34^	BDeu (n = 31) vs. HC (n = 27)	HDS, YMRS	AuT (process) RAT (process)	BDeu < HC
Pei-Chi Tu ^35^	BDeu (n = 59) vs. HC (n = 56)	HDRS-17, YMRS	ATTA (process) CWRAT (process)	BDeu = HC
Johnson ^38^	BDeu (n = 62) vs. HC (n = 50)	ASRM, BDI, MHRSD, YMRS	CAQ (person) UUT (process)	BDeu = HC
Soeiro-de-Souza ^39^	BDeu (n = 42) + BDm (n = 44) + BDd (n = 33) vs. HC (n = 97)	MADRS, YMRS	BWAS (person)	BDeu + BDm + BDd < HC
Soeiro-de-Souza ^40^	BDm (n = 41) + BDd (n = 25) vs. HC (n = 78)	MADRS, YMRS	BWAS (person)	BDm + BDd > HC
Rybakowski ^41^	BDeu (n = 40) vs. HC (n = 48)	HDRS-17, YMRS	BIS (process) RAS (person)	BIS: BDeu > HC RAS: BDeu = HC
Santosa ^42^	BDeu (n = 49) vs. creative controls (n = 32) vs. HC (n = 47)	BDI	ACL-CPS (person) BWAS (person) TTCT-F (process) TTCT-V (process)	BWAS: BDeu and creative controls > HC TTCT-F: BD = creative controls = HC TTCT-V and ACL-CPS: BD = creative controls = HC
Simeonova ^43^	BD (parents) (n = 40) vs. HC (parents) (n = 18) BD (BD’s children) (n = 20) vs. HC (HC’s children) (n = 18)	CDRS-R, YMRS	BWAS (person)	BD (parents) = HC (parents) BD (BD’s children) > HC (HC’s children)

ACL-CPS = Adjective Check List Creative Personality Scale; ASRM = Altman Self-Rating Mania Scale; ATTA = Abbreviated Torrance Test for Adults; AuT = Alternate Uses Test; BD = bipolar disorder; BDd = BD in depression; BDeu = BD in euthymia; BDI = Beck Depression Inventory; BDm = BD in mania; BIS = Berlin Intelligence Structure Test; BWAS = Barrow Welsh Art Scale; CAQ = Creative Achievement Questionnaire; CDRS-R = Children’s Depression Rating Scale-Revised; CWRAT = Chinese Word Remote Associates Test; HC = healthy controls; HDRS-17 = Hamilton Depression Rating Scale; HDS = Hamilton Depression Scale; MADRS = Montgomery-Asberg Depression Rating Scale; MHRSD = Modified Hamilton Rating Scale for Depression; RAS = Revised Art Scale; RAT = Remote Associates Test; TTCT-F = Torrance Tests of Creative Thinking – Figural; TTCT-V = Torrance Tests of Creative Thinking – Verbal; UUT = Unusual Uses Task; YMRS = Young Mania Rating Scale.

**Table 2:** Studies comparing BD patients with individuals with other mental disorders

Study	Design and samples	Clinical assessment instruments	Creativity assessment instruments	Results
Santosa ^42^	BDeu (n = 49) vs. MDD (n = 25) vs. creative controls (n = 32) vs. HC (n = 47)	BDI	ACL-CPS (person) BWAS (person) TTCT-F (process) TTCT-V (process)	BWAS: BDeu and Creative Controls and MDD > HC TTCT-F: creative controls > MDD TTCT-V and ACL-CPS: BD = creative controls = HC = MDD
Simeonova ^43^	BD (BD’s children) (n = 20) vs. ADHD (BD’s children) (n = 20)	CDRS-R, YMRS	BWAS (person)	BD = ADHD
Forgeard ^44^	BDeu (n = 16) + BDm (n = 1) + BDd (n = 43) vs. anxiety + psychosis + PTSD + SUD (133)	CESD-10, RSS	Creative Thinking Task (process)	BDeu + BDm + BDd = anxiety + psychosis + PTSD + SUD
Richards ^45^	BD (n = 17) vs. cyclothymia’s relatives (n = 11) + cyclothymia (n = 16)	Medical records	LCS (product)	BD < cyclothymia + cyclothymia’s relatives

ACL-CPS = Adjective Check List Creative Personality Scale; ADHD – attention deficit hyperactivity disorder; ASRM = Altman Self-Rating Mania Scale; ATTA = Abbreviated Torrance Test for Adults; AuT = Alternate Uses Test; BD = bipolar disorder; BDd = BD in depression; BDeu = BD in euthymia; BDI = Beck Depression Inventory; BDm = BD in mania; BIS = Berlin Intelligence Structure Test; BWAS = Barrow Welsh Art Scale; CAQ = Creative Achievement Questionnaire; CDRS-R = Children’s Depression Rating Scale-Revised; CESD-10 = 10-Item Center for Epidemiological Studies Depression Scale; CWRAT = Chinese Word Remote Associates Test; HC = healthy controls; HDRS-17 = Hamilton Depression Rating Scale; HDS = Hamilton Depression Scale; LCS = Lifetime Creativity Scales; MADRS = Montgomery-Asberg Depression Rating Scale; MDD = major depressive disorder; MHRSD = Modified Hamilton Rating Scale for Depression; PTSD = posttraumatic stress disorder; RAS = Revised Art Scale; RAT = Remote Associates Test; RRS = Ruminative Responses Scale; SUD = substance use disorders; TTCT-F = Torrance Tests of Creative Thinking – Figural; TTCT-V = Torrance Tests of Creative Thinking – Verbal; UUT = Unusual Uses Task; YMRS = Young Mania Rating Scale.

Eight of these 10 studies conducted comparisons with healthy individuals and one study included two groups of healthy controls, ^42^ one group comprising individuals considered creative, that is, students of the creative writing, fine arts, and product design courses at Stanford University. The results of the comparisons between BD patients and healthy controls were quite heterogeneous. The highest levels of creativity were observed in BD patients in two studies ^40 , 43^ and in healthy controls in two others. ^34 , 39^ There was no difference between the two groups in two studies. ^35 , 38^ In the remaining two studies, ^41 , 42^ depending on the instrument used, BD patients were considered more creative or there was a tie. In the study in which the group of so-called “creative controls” was included, depending on the instrument used, these individuals were more creative than BD patients or there was a tie. ^42^

Four of the 10 studies performed comparisons with individuals who suffered from other mental disorders: major depressive disorder (MDD), ^42^ attention deficit hyperactivity disorder (ADHD), ^43^ cyclothymia, ^45^ and various mental disorders (anxiety disorders, post-traumatic stress disorder, psychoses, and substance use disorders). ^44^ In the study with ADHD, both ADHD patients and those with BD had parents who suffered from BD. That study examined creativity in children of bipolar parents in order to clarify possible intergenerational transmission. Bipolar parents and their children with BD or ADHD were compared to healthy parents and their healthy children. There was no significant difference in the overall results between bipolar adults and healthy adults. However, children with BD and children with ADHD were significantly more creative than healthy children. Finally, in the study with cyclothymia, the group of patients with this diagnosis also included their family members. There were no differences in performance in the creativity tests when patients with BD were compared to the group of patients with MDD, ^42^ to patients with various mental disorders, ^44^ or to patients with ADHD. ^43^ However, the performance of patients with BD was inferior to a group consisting of patients with cyclothymia and their families. ^45^

Four of the 12 studies compared different mood states with each other ^36 , 37 , 40 , 41^ ( Table 3 ). All four of these included a group of patients in mania and a group of patients in depression; two also included a group of patients in mixed states, ^36 , 37^ and one had a group of patients in euthymia. ^37^ The severity of affective symptoms was assessed, but only to identify mood states, that is, mania, depression, or euthymia. None of the four studies investigated a possible correlation between the severity of symptoms and the level of creativity.

**Table 3:** Studies comparing bipolar patients in different states of the disease

Study	Design and samples	Clinical assessment instruments	Creativity assessment instruments	Results
Soeiro-de-Souza ^36^	BDm (n = 20) vs. BDd (n = 26) vs. BDmx (n = 21)	MADRS, YMRS	BWAS (person)	BDm and BDmx > Bdd BDm > BDmx
Miller ^37^	BDh + BDm (n = 68) vs. BDd (n = 167) vs. BDeu (n = 94) vs. BDmx (n = 68)	ASRM, DASS-21	CDQ-R (person)	BDd = Bdmx BDd < BDh + BDm + BDmx +BDeu BDh + BDm > Bdmx BDh + BDm = BDeu
Soeiro-de-Souza ^40^	BDm (n = 41) vs. BDd (n = 25)	MADRS, YMRS	BWAS (person)	BDm > BDd
Rybakowski ^41^	BDm (n = 22) vs. BDd (n = 18)	HDRS-17, YMRS	BIS (process) -RAS (person)	BDm > BDd

*A* SRM = Altman Self-Rating Mania Scale; BD = bipolar disorder; BDd = BD in depression; BDeu = BD in euthymia; BDh = BD in hypomania; BDm = BD in mania; BDmx = BD in mixed state; BIS = Berlin Intelligence Structure Test; BWAS = Barrow Welsh Art Scale; CDQ-R = Creativity Domain Questionnaire-Revised; DASS-21 = Depression Anxiety Stress Scale; HDRS-17 = Hamilton Depression Rating Scale; MADRS = Montgomery-Asberg Depression Rating Scale; RAS = Revised Art Scale; YMRS = Young Mania Rating Scale.

In these four studies, patients in bipolar depression performed worse on assessments of creativity than patients in mania. ^36 , 37 , 40 , 41^ In one of these four studies, patients in bipolar depression also had lower results in the comparisons with patients in euthymic and mixed states. ^37^ Patients in mania (or hypomania) were more creative than patients in mixed states in two studies. ^36 , 37^ Finally, in one study, patients in mania (or hypomania) were not distinguished from euthymia patients. ^37^

## Discussion

The objectives of this systematic review were to investigate the relationship between BD and creativity and whether creative capacity varies depending on mood state. According to the results found, it is not possible to say that BD patients are more creative than healthy controls or than patients with other mental disorders. In the comparisons between the different stages of BD, we observed that patients in depression or in mixed states had the worst performances, however patients in mania did not perform better than patients in euthymia.

The conflicting results reflect the methodological weaknesses often found in research on creativity. ^27^ Most studies did not define the concept of creativity and none of them considered multidimensionality. Although they were investigating a multidimensional concept, no study evaluated more than two aspects of creativity, and the majority evaluated only one. The most studied aspects were the process and the person. Since the results were heterogeneous, it is not possible to say that BD patients are more creative in any of these aspects.

Humor and motivation are important elements for creativity. In the depressive phase of BD, most mental activity is inhibited. Patients often complain of slowness of thought, confusion, and an inability to concentrate. They feel inadequate and useless. ^69^ Thus, it is to be expected that there would be a decrease in productivity and creativity. On the other hand, in hypomania, the increased speed of thoughts can contribute to creative production, increasing the number of thoughts and producing unique ideas and associations. ^70^ Due to the attenuated symptoms, it can be assumed that it is during hypomania that people with BD would obtain the greatest benefits from their creativity. In contrast, in mania, it is very common for patients to produce with reduced quality or to give up their creative tasks before completing them. ^70^ In this mood state, people with BD can easily be distracted by any stimuli and their thoughts can be accelerated, becoming disconnected and inconsistent with reality. ^69^

The results of the studies we found went against some of our expectations. One explanation for this may be the fact that none of the studies included a sample of BD patients made up solely of individuals with hypomania. Moreover, creativity was assessed cross-sectionally in practically all of the studies, even though it would only be with all patients in the hypomania mood state that BD would be associated with better performance than healthy controls and patients with other mental disorders. In view of this, the results of the study in which individuals with cyclothymia were more creative than those with BD are not surprising. Since cyclothymia is an “attenuated” BD, it could count on some of the advantages of BD, such as creativity, without the most serious negative consequences. When the different mood states of BD were compared, patients in depression were less creative, as expected, but because none of the studies separated patients in mania from those in hypomania, it was not possible to confirm whether the latter mood state is in fact associated with an increase in creativity.

Many creative individuals who suffer from BD consider emotional turbulence essential to their identities and worry that psychiatric treatment compromises their ability to create. ^69^ There are various ways in which the medications used to treat BD can alter characteristics important for creativity, such as motivation to achieve goals, disinhibition, novelty seeking, and the ability to associate ideas. ^71^ There is evidence that treatment of people with BD with lithium can decrease associative productivity ^72 , 73^ and artistic creativity. ^74^ Measures of memory and motor performance significantly improve when lithium is withdrawn and decrease when treatment is restarted. ^73^ Even if the patient is not an artist or an academic, creativity should be taken into account by clinicians. ^71^ Doctors should be aware of the side effects of medications on the creative process and, whenever possible, reduce doses. ^69^

Creativity depends on a set of mental operations applied to existing knowledge, through cognition. ^75^ Creative people stand out for their greater ability to organize their ideas effectively, selecting those that are useful and discarding the others. ^76^ To achieve this goal, the main cognitive skills involved in the creative process are attention, memory, and executive functions. It is the combination of these processes that makes a person able to maintain and manipulate information in working memory, suppress irrelevant information, and change strategies as needed. ^77^ People with BD can present widespread and diffuse cognitive impairment in all mood states, including euthymia. ^69^ The most prominent deficits involve executive function, attention, verbal memory, and non-verbal memory, ^77^ which are precisely the skills essential to the creative process. Thus, in theory, people with BD can only develop their creativity if they do not have severe cognitive impairment. None of the articles selected investigated the relationship between cognition and creativity in BD. This could be an important research topic for future studies.

Given the results we found, it would be interesting to find studies on creativity in other mental disorders. Like BD, schizophrenia is often associated with creativity. ^78^ Considering that originality is one of the criteria for defining this construct, people with schizophrenia could really be called creative, since their thoughts are more likely to be different, new, or original. ^76^ However, although this mental disorder can facilitate production of original responses, it does not favor production of appropriate responses. ^79^ A meta-analysis ^80^ indicates that mild expression of schizophrenia symptoms can be positive for creativity, but that the complete presentation of the disorder impairs this ability. Similarly, a meta-analysis on autism ^81^ clarifies that patients with this diagnosis, compared to healthy controls, demonstrate a greater ability to offer original responses and responses rich in details, but offer smaller quantities of responses and are less able to produce semantically different ideas. The authors conclude that there are fewer creative individuals among people with autism than in the general population. ^81^ Although BD patients also have cognitive impairment, the positive effects could contribute to creativity. On the other hand, it is possible that the emotional dullness found in both schizophrenia and autism represents yet another difficulty in exercising this ability.

Some limitations related to our study must be mentioned We only found 12 studies eligible for our review. The samples in these studies were small and comprised clinical patients, therefore, severe patients. Thus, the results may not reflect the average experience of a person with BD. Patients with BD I, II, and unspecified BD were combined in the same groups. There were no groups with only hypomanic patients. There was also no uniform concept of creativity. The use of different instruments made it difficult to evaluate the results. Future research would benefit from definition of the concept of creativity and investigation of all four aspects of the concept. The environment – the only concept not investigated in any of the studies – can offer important information for understanding the relationship between creativity and BD.

Unlike other systematic reviews, we only selected studies that employed operational criteria for diagnosis of BD and used objective instruments for assessment of creativity. On the other hand, in clinical practice, we often hear reports from patients who have been involved in many activities and have been productive during periods of hypomania and mania. A phase of increased creativity may be indicative of a new episode of the disease. In relation to depression, the reasoning would be the other way around. In this mood state, patients are less creative and productive. Therefore, reduced activity may indicate a new depressive episode. If the association between creativity and BD is confirmed, this characteristic could become more valued and contribute to reducing stigma against patients.

## Conclusion

The idea of a relationship between BD and creativity is based on the study of biographies of successful artists and on the high prevalence rates of this mental disorder among living artists when modern diagnostic criteria are applied. For the time being, few clinical studies have been conducted that compared, in terms of creative capacity, patients suffering from BD with healthy controls or with patients with other psychiatric diagnoses. Even rarer were studies that compared the different mood states in terms of creativity. The results of these clinical studies indicate that, in BD, depression is associated with worse performance in creativity tests. However, it was not possible to prove that the hypomanic phase progresses with an increase in creativity, which is probably due to the fact that all of the studies that included patients with hypomania mixed them with patients in mania. In addition, it was not possible to corroborate the idea that individuals with BD are more creative than individuals without psychiatric diagnoses or than patients suffering from other mental disorders, which may be related to the choice of cross-sectional rather than longitudinal designs in virtually all of the clinical studies.
